# The Red Cell Distribution Width to Albumin Ratio: A Novel Prognostic Indicator in Hepatitis B Virus-Related Hepatocellular Carcinoma

**DOI:** 10.7150/ijms.103125

**Published:** 2025-01-01

**Authors:** Maoqing Tan, Ruolan You, Danni Cai, Jin Wang, Wei Dai, Rong Yang, Dongliang Li, Huifang Huang

**Affiliations:** 1 Central Laboratory, Fujian Medical University Union Hospital, Fuzhou, Fujian, 350001, China.; 2 Department of Hepatobiliary Disease, Fuzong Clinical Medical College of Fujian Medical University (900TH Hospital of Joint Logistics Support Force), Fuzhou, Fujian, 350025, China.; 3 Follow-Up Center, Fujian Medical University Union Hospital, Fuzhou, Fujian, 350001, China.

**Keywords:** Albumin, Red cell distribution width, Hepatocellular carcinoma, Prognosis, Net reclassification index

## Abstract

**Background:** The prognostic significance of the red blood cell distribution width to albumin ratio (RAR) spans various diseases, yet its utility as a biomarker for hepatitis B virus-related hepatocellular carcinoma (HBV-HCC) remains unclear.

**Methods:** We retrospectively studied 1,413 patients with HBV-HCC. Receiver operating characteristic curves identified optimal RAR cut-offs, stratifying patients into H-RAR and L-RAR groups. Propensity score matching helped balance baseline characteristics. We further evaluated the incremental predictive value of RAR by incorporating it into established conventional models.

**Results:** Overall, 906 patients with HBV-HCC were enrolled (H-RAR group, 600 (66.2%); L-RAR group, 306 (33.8%)). After propensity score matching, 209 patients were included in each group with balanced baseline characteristics (all *p* > 0.05). RAR demonstrated superior prognostic discrimination compared to red blood cell distribution width, albumin, total bilirubin, and Child-Pugh scores alone, with an area under the curve (AUC) of 0.751. The risk of all-cause mortality increased progressively within a specific RAR range. High RAR was identified as an independent risk factor for long-term overall survival in patients with HBV-HCC (hazard ratio = 1.707, 95% confidence interval [CI]: 1.338-2.176). Stratification by tumour stage revealed substantially lower overall survival for H-RAR than for L-RAR across Tumour, Node, Metastasis I-IV stages. Incorporating RAR into traditional HCC staging systems substantially improved the ability to predict overall mortality risk.

**Conclusion:** RAR is a novel and valuable prognostic indicator for patients with HBV-HCC.

## Introduction

Hepatocellular carcinoma (HCC) is the predominant form of primary liver cancer, constituting 75%-85% of all liver cancer cases. It is the second most lethal tumour globally and the third leading cause of cancer-related deaths 1, 2. Surveys indicate that chronic hepatitis B virus (HBV) infection is the primary cause of HCC in the Chinese population, responsible for approximately 80% of liver cancer cases 3. Although there are multiple treatment options for HCC, including surgical resection, liver transplantation, and radiofrequency ablation, less than 30% of patients with HCC qualify for these therapies due to liver dysfunction and tumor metastasis 4-6. Furthermore, HCC demonstrates considerable heterogeneity and drug resistance, considerably diminishing the efficacy of local and systemic treatments 7. Identifying independent prognostic indicators is crucial for the early detection of high-risk patients with HCC, enabling personalised treatment strategies and improving clinical outcomes 6.

Red blood cell distribution width (RDW) is an accessible parameter that reflects the heterogeneity of red blood cell volume. Traditionally employed to diagnose and classify anaemia 8, 9, recent studies have highlighted its importance in various malignant tumours, including colorectal cancer 10, gastric cancer 11, oesophageal cancer 12, prostate cancer 13, and acute myeloid leukaemia 14. Interestingly, the RDW/platelet ratio has been identified as an effective and promising non-invasive tool for predicting the timing and manner of decompensation in patients with metabolic dysfunction-associated steatotic liver disease-induced cirrhosis 15. Moreover, several studies have demonstrated that RDW is a potential predictor of survival in patients with HCC 16, 17. Our previous research has also confirmed that elevated RDW is associated with poor prognosis in patients with HBV-HCC, further highlighting RDW as a valuable prognostic marker in this population 18.

Human serum albumin (ALB), the most abundant plasma protein, is mainly produced and secreted by the liver. It has various biological functions, including regulation of plasma osmotic pressure, immune responses, inhibition of oxidative stress, and prevention of endothelial cell apoptosis 7. Evidence confirms the prognostic value of serum ALB levels in patients with HCC: lower ALB levels are correlated with larger HCC tumours, whereas elevated levels inhibit tumour growth, invasion, and migration 19-22.

Previous studies have suggested the ratio of RDW to ALB (RAR) as a composite parameter reflecting both nutritional and inflammatory status, showing strong prognostic associations with various conditions such as diabetes, coronary artery disease, sepsis, acute kidney injury, and cancer 9, 23-26. However, specific data on the prognostic value of RAR in patients with HBV-HCC are lacking. The current study investigates the potential association between RAR and the prognosis of patients with HBV-HCC and explores the impact of RAR on overall mortality in this population.

## Materials and Methods

### Participants

**Figure 1:**
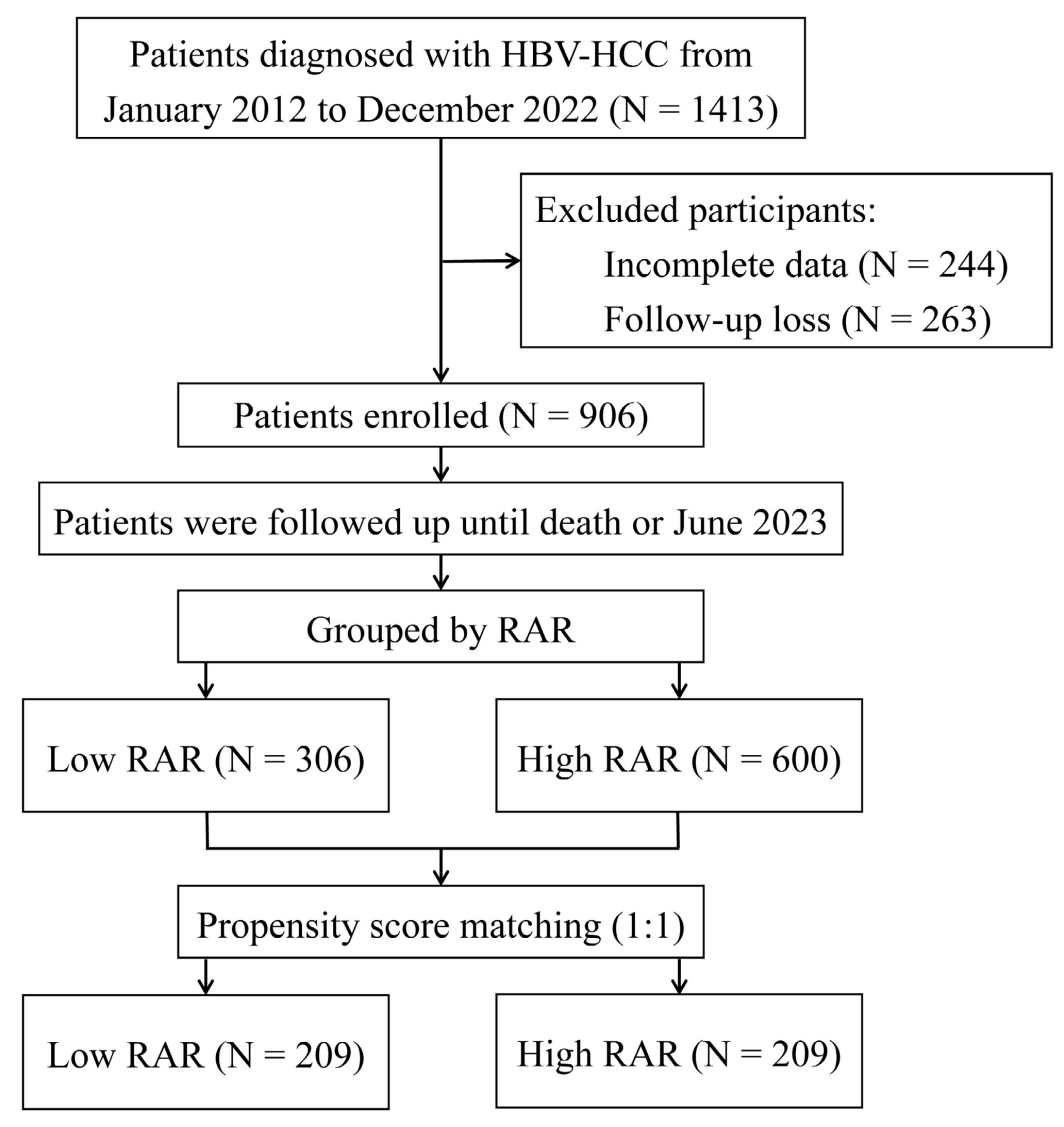
Flow chart of the study. HBV-HCC, hepatitis B virus-related hepatocellular carcinoma; RAR, red blood cell distribution width to albumin ratio.

From January 2012 to December 2022, a total of 1,413 participants with HBV-HCC first diagnosed at the Fuzong Clinical Medical College of Fujian Medical University were retrospectively examined. The inclusion criteria were: (1) confirmation of HCC through radiological or pathological diagnosis; (2) presence of hepatitis B surface antigen for more than 6 months; and (3) no prior anti-tumour therapy. Conversely, the exclusion criteria were as follows: (1) concurrent infection with other hepatitis viruses; (2) coexistence of other tumours; (3) gastrointestinal bleeding within the past 6 months; (4) co-occurrence of haematologic disorders; and (5) incomplete clinical or follow-up information. The specific research workflow is illustrated in Figure 1.

The Ethics Committee of Fuzong Clinical Medical College of Fujian Medical University approved this study (Approval Number: 2022-014). Written authorisation was not required, as all patient records were anonymised prior to the analysis.

### Demographic and clinical data

We retrospectively retrieved patient histories and baseline characteristic data, including demographic and laboratory parameters, from the medical record system. This study focused on assessing the overall survival (OS) of individuals diagnosed with HBV-HCC. OS, measured from radiological confirmation, spanned from hospital admission until either all-cause mortality or the last follow-up, with follow-up concluding in June 2023.

### Propensity score matching

To mitigate retrospective study biases, this study used a 1:1 propensity score matching (PSM) method between high RAR and low RAR groups using nearest neighbour matching without replacement, with a calliper set at 0.1. Variables at baseline showing imbalances (*p* < 0.05) were included in the PSM analysis, comprising age; neutrophil count; red blood cell count; haematocrit; HBV DNA; cirrhosis; maximum tumour diameter; tumour number; Tumour, Node, Metastasis (TNM) staging; and initial treatment approach.

### Statistical analysis

The Kolmogorov-Smirnov test was used to examine the normality of variables. All continuous variables showed non-normal distributions and are presented as the median and interquartile range (IQR). Categorical data were expressed as absolute counts and percentages. Differences between the two groups were compared using the Mann-Whitney U test for continuous variables and the chi-square test for categorical variables. Receiver Operating Characteristic curve (ROC) analysis was used to compare the predictive abilities of RDW, ALB, Child-Pugh scores, total bilirubin (TBIL), and RAR for overall mortality risk in patients with HBV-HCC, determining the optimal cutoff value for RAR. Spearman's correlation coefficient analysis was conducted to assess correlations between variables, whereas restricted cubic spline (RCS) analysis was used to evaluate potential nonlinear relationships between RAR and overall death risk. The Kaplan-Meier method was applied for survival curve estimation, and the log-rank test was used for comparisons. Cox regression analysis was used for univariate analysis of variables potentially affecting OS, followed by multivariate analysis including variables with *p*-values < 0.05 from the univariate regression analysis to identify independent prognostic factors affecting OS. Additionally, the concordance index (C-index), integrated discrimination improvement (IDI), net reclassification improvement (NRI), and time-dependent ROC curve were used to evaluate the improvements in model discrimination, risk reclassification, and time-dependent predictive accuracy after integrating RAR into traditional prognostic models. The likelihood ratio chi-square test was used to compare the goodness of fit between nested models. Significant differences were defined at *p* < 0.05.

## Results

### Patient baseline characteristics

A total of 906 patients were included in this study, with a median age of 54 years (IQR: 46-63). Male patients accounted for 86.2% of the cohort (781/906). The median follow-up duration was 54 months (95 CI: 50-58 months), with 1-year, 3-year, and 5-year overall survival rates of 59.4%, 35.1%, and 25.4%, respectively (Fig. S7). In this study, 70.6% (640/906) of patients underwent surgical treatment, with a median survival time of 34 months (95% CI: 29-39 months). In contrast, 29.4% (266/906) received non-surgical treatment, with a median survival time of only 4 months (95% CI: 3—5 months). Table S1 provides an overview of the baseline characteristics of the entire population, including demographic data, laboratory results, tumor characteristics, and treatment approaches. Based on the optimal cutoff value for survival-based RAR (0.3217%/[g/L]), patients were classified into the H-RAR group (66.2%, 600/906) and the L-RAR group (33.8%, 306/906). The H-RAR group exhibited several notable characteristics: advanced age, elevated neutrophil levels, positive HBV DNA status, a high prevalence of cirrhosis, larger tumour diameters, and more advanced tumour stages. Moreover, this group showed reduced red blood cell counts and haematocrit levels, along with a lower proportion of patients undergoing surgical treatment (all *p* < 0.05; Table 1). Following PSM, 209 patients were included in each group, demonstrating balanced baseline characteristics between the two groups (all *p* > 0.05, Table 1).

### Comparison of RAR with individual parameters

**Figure 2:**
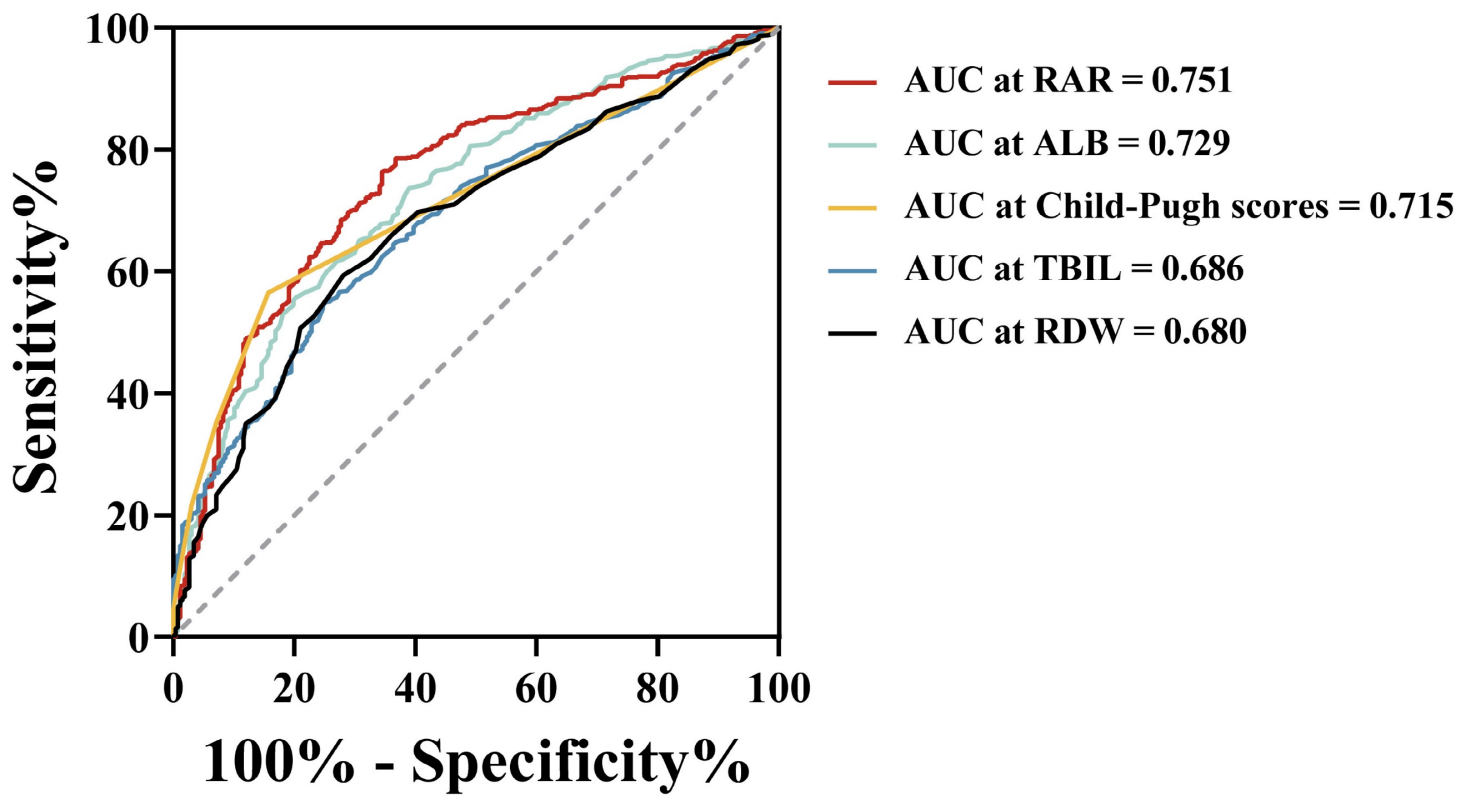
ROC curves of RDW, ALB, TBIL, Child-Pugh scores and RAR for predicting all-cause mortality in the overall study population. ROC, receiver operating characteristic; AUC, area under curve; RDW, red cell distribution width; ALB, albumin; TBIL, total bilirubin; RAR, red blood cell distribution width to albumin ratio.

Figure 2 illustrates the discriminative ability of the combined index RAR compared with the individual indices RDW, ALB, TBIL, and Child-Pugh scores for predicting prognosis in patients with HBV-HCC. The results show that RAR demonstrated superior predictive performance compared to these individual indicators. In the entire cohort, the area under the ROC curve (AUC) for RDW was 0.680, with a sensitivity of 59.3% and a specificity of 71.9%. ALB exhibited an AUC of 0.729, with a sensitivity of 55.7% and specificity of 79.8%. For TBIL and the Child-Pugh scores, the AUCs were 0.686 and 0.715, with corresponding sensitivities of 54.9% and 56.5%, and specificities of 74.9% and 84.3%, respectively. Notably, RAR demonstrated the highest AUC of 0.751, with improved sensitivity (78.6%) and moderate specificity (63.3%).

### Correlation and dynamic mortality risk for RAR

Figure S1 shows the correlation between RAR and other parameters. RAR was positively correlated with C-reactive protein (CRP; r = 0.450), TBIL (r = 0.460), Child-Pugh scores (r = 0.706), and maximum tumour diameter (r = 0.312), but negatively correlated with LYM (r = -0.388) (all *p* < 0.0001). However, there was no correlation between RAR and age (r = 0.098).

The RCS in Figure S2 shows the dynamic mortality risk changes of RAR. Within a specific range, the risk of all-cause mortality showed a gradual upward trend as RAR increased (*p* < 0.001).

### Unadjusted and PSM-adjusted survival analysis

In the entire cohort, the OS of the H-RAR group was significantly lower than that of the L-RAR group (5-year OS was 4.2% and 24.2%, respectively, *p* < 0.05) (Fig. S3). Multivariate Cox regression analysis showed that RAR was a long-term independent prognostic factor for OS (hazards ratio [HR] = 1.567, 95% confidence interval [CI]: 1.251-1.964) (Table S2). Subsequent stratified analysis across TNM stages I-IV indicated a considerable difference in OS between the H-RAR and L-RAR cohorts (all *p* < 0.05; Fig. S4). A difference in OS was observed at different survival time points (1, 2, 3, and 5 years) (all *p* < 0.05; Fig. S5).

**Figure 3:**
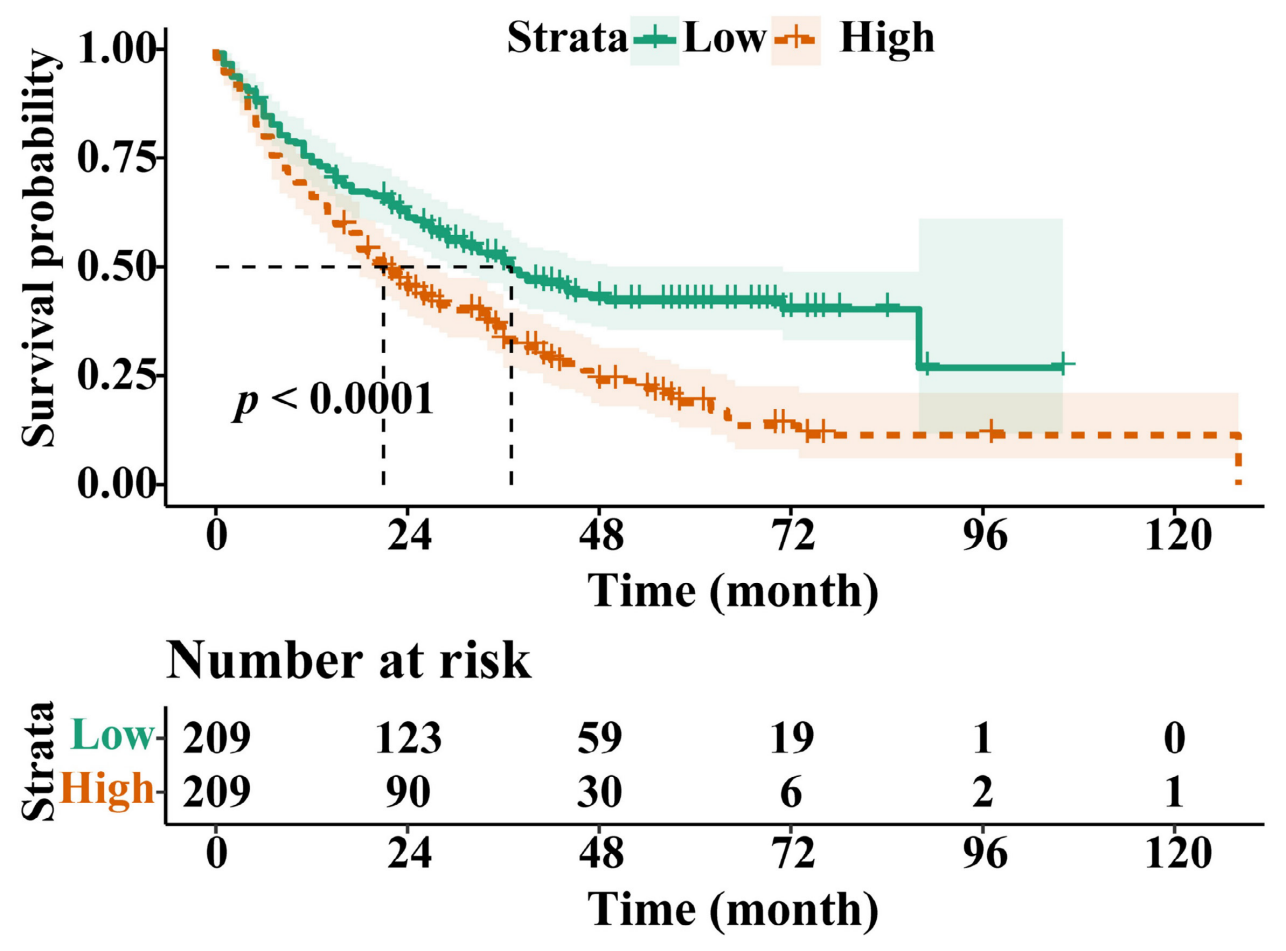
Overall survival stratified by red blood cell distribution width to albumin ratio after propensity-score matching in the entire cohort.

**Figure 4:**
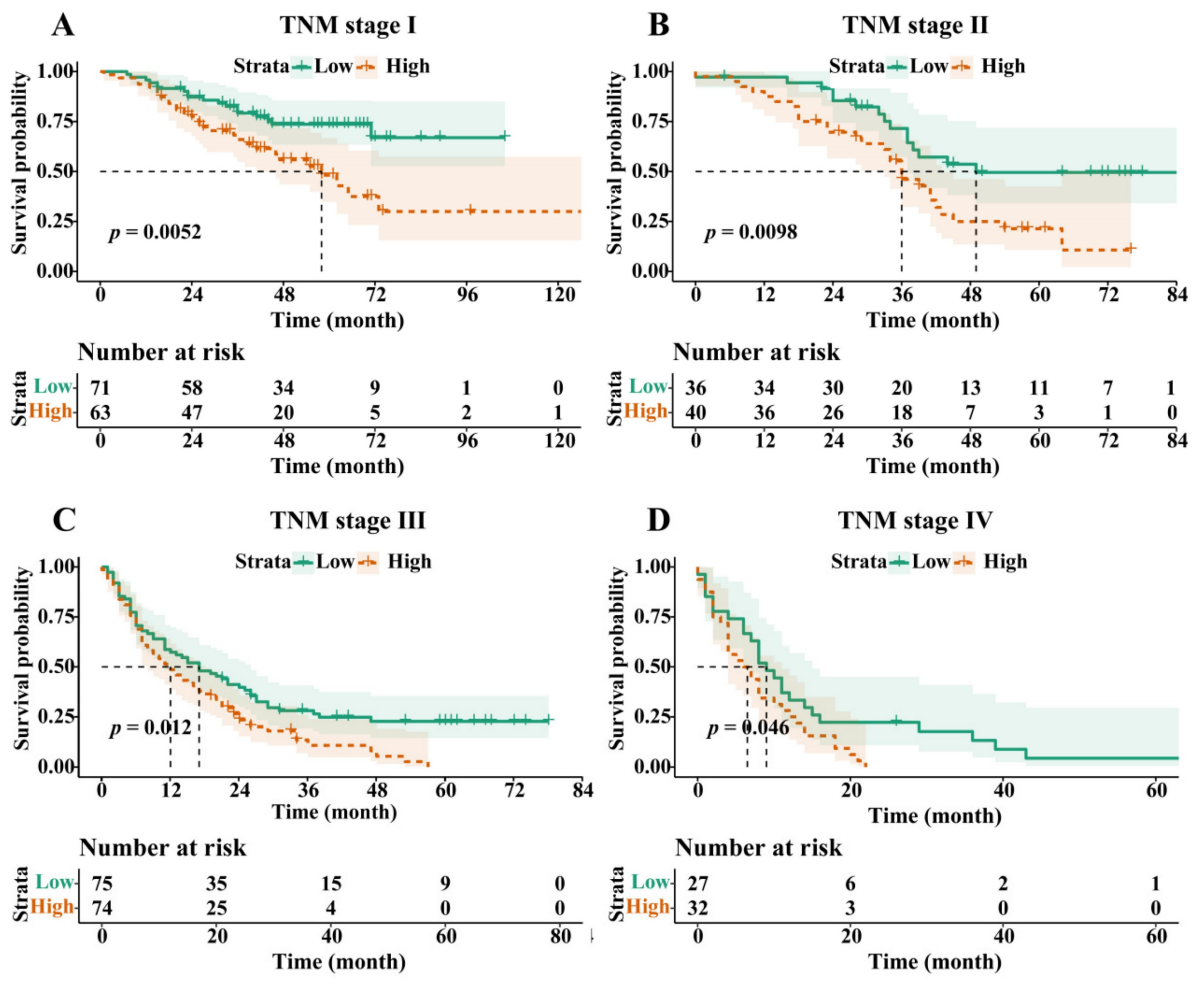
Overall survival stratified by red blood cell distribution width to albumin ratio in Tumour, Node, and Metastasis (TNM) stage I (A), stage II (B), stage III (C), and stage IV (D) cohorts after propensity-score matching.

Following PSM, the OS of the H-RAR group remained significantly lower than that of the L-RAR group (5-year OS was 6.2% and 23.0%, respectively, *p* < 0.05, Fig. 3). Even for patients at different TNM stages, the difference in OS between the H-RAR and L-RAR groups persisted, consistent with the results before PSM (all *p* < 0.05, Fig. 4). However, when stratifying survival time, the one-year OS between the H-RAR and L-RAR groups after PSM was similar (*p* = 0.081), whereas the ≥ two-year OS of the H-RAR group was lower (*p* < 0.05; Fig. S6). After univariate analysis, variables such as age, sex, white blood cell count, neutrophil count, lymphocyte count, monocyte count, platelet count, RAR, Child-Pugh grade, HBV DNA, alpha-fetoprotein (AFP), TNM stage, maximum tumour diameter, tumour number, and initial treatment regimen were associated with OS (all *p* < 0.05). However, subsequent multivariate Cox regression analysis indicated that only RAR (HR = 1.571, 95% CI: 1.202-2.053, *p* = 0.001), Child-Pugh grade (grade C [HR = 8.442, 95% CI: 3.353-21.255, *p* < 0.001]), TNM stage (stage III [HR = 2.988, 95% CI: 1.797-4.969, *p* < 0.001], stage IV [HR = 4.411, 95% CI: 2.440-7.974, *p* < 0.001]), maximum tumour diameter (HR = 1.053, 95% CI: 1.016-1.092, *p* = 0.005), and initial treatment mode (HR = 2.099, 95% CI: 1.488-2.960, *p* < 0.001) were identified as independent prognostic factors for OS in patients with HBV-HCC (Table 2).

### The incremental predictive value of RAR

RAR was further incorporated into traditional prognostic models (BCLC, TNM, Child-Pugh grade, CLIP, ALBI) to assess its predictive ability for all-cause mortality. The results indicate that all models, except for the ALBI model, exhibited significant improvements in predictive performance following the incorporation of RAR. Specifically, the C-index for the BCLC model increased from 0.745 to 0.757, with an IDI increase of 5.0% (*p* < 0.001) and a NRI increase of 36.1% (*p* < 0.001). The time-dependent AUC improved from 0.815 to 0.851. For the TNM model, the C-index rose from 0.734 to 0.749, the IDI increased by 6.1% (*p* < 0.001), and the NRI also showed a 36.1% enhancement (*p* < 0.001), with the time-dependent AUC advancing from 0.787 to 0.840. The Child-Pugh grade and CLIP model similarly demonstrated significant improvements, with C-indices rising from 0.554 and 0.533 to 0.585 and 0.566, respectively. The IDI increased by 4.1% and 5.0%, while the NRI improved by 34.1% and 36.1%, respectively. The time-dependent AUCs increased from 0.575 and 0.572 to 0.698 and 0.683. Furthermore, the likelihood ratio chi-square test further confirmed the significant enhancement in model fitting, with χ² values for the BCLC, TNM, Child-Pugh grade, and CLIP models being 21.102, 28.497, 9.499, and 12.587, respectively (Table 3, Figure S8).

## Discussion

RAR, a novel indicator combining RDW and ALB, has emerged as a valuable prognostic marker for diverse inflammatory diseases. Huang *et al.* 23 demonstrated a correlation between RAR and carotid plaque formation in patients with coronary heart disease (OR = 1.23; 95% CI: 1.08-1.39). Seo *et al.* identified that elevated RAR levels were strongly associated with increased 90-day mortality, increased intensive care unit stay rates, and prolonged intensive care unit stays for burn victims.

Moreover, RAR exhibits potential in forecasting cancer prognoses 26. To the best of our knowledge, this is the first study to report the potential prognostic value of RAR in patients with HBV-HCC. Our findings suggest that RAR has robust predictive value for patients with HBV-HCC across all tumour stages and may offer superior predictive accuracy for long-term OS.

Herein, we retrospectively analysed 906 patients with HBV-HCC and used PSM to minimise bias. A comparison of ROC curves indicated that the combined parameter RAR, was better than RDW, ALB, Child-Pugh scores, or TBIL in discriminating all-cause mortality, suggesting the potential prognostic value and advantages of RAR in patients with HBV-HCC. In addition, RAR was strongly correlated with inflammatory biomarkers, such as CRP and LYM, and positively correlated with TBIL, Child-Pugh scores, and maximum tumour diameter, suggesting that RAR has the potential to reflect inflammatory status, liver function, and tumour burden. RCS analysis also showed that the risk of all-cause mortality gradually increased with an increase in RAR within a certain range, further suggesting that RAR is associated with the prognosis of patients with HBV-HCC. Subsequent survival analysis showed that patients in the H-RAR group had worse OS; RAR was an independent prognostic factor for long-term OS in patients with HBV-HCC. Similar results were observed by stratified analysis of tumour stages. The H-RAR and L-RAR groups had similar 1-year OS, whereas the ≥ 2-year OS was markedly reduced in the H-RAR group after PSM, suggesting that RAR was advantageous in predicting long-term OS in patients with HBV-HCC. Further studies are warranted to confirm this. When RAR was added to the traditional prognostic model, the new model substantially improved the ability to predict all-cause mortality. This further suggests that RAR is an important predictor of prognosis in patients with HBV-HCC.

While the precise mechanisms through which HBV-HCC influences RAR have not been fully elucidated, there are compelling indications that it may be associated with chronic inflammation and malnutrition 27, 28. Previous studies have shown that elevated RDW is associated with adverse outcomes of many inflammatory diseases, such as pneumonia 29, sepsis 30, and acute respiratory distress syndrome 31. Chronic inflammation may alter erythropoiesis through pro-inflammatory cytokines, such as interleukin-1, tumour necrosis factor-α, and interferon-γ 32. In vitro studies have also shown that these cytokines can effectively suppress erythroid colony formation, especially interferon-γ, which promotes the apoptosis of erythroid progenitor cells, thus antagonising the anti-apoptotic effect of erythropoietin 33. In addition, in the inflammatory state, excessive reactive oxygen species may affect the deformability of red blood cells by changing membrane proteins and membrane elastic networks, whereas an increase in nitric oxide may also reduce the deformability of red blood cells 34. In summary, malignant tumours induce a wide range of systemic inflammation by secreting cytokines and releasing tumour degradation products, inhibiting erythropoiesis, and accelerating apoptosis 35. Malignant tumours also induce cachexia and malnutrition, resulting in a long-term deficiency of various trace elements and vitamins, such as iron, folic acid, and vitamin B12, eventually increasing the heterogeneity of red blood cell volume and RDW levels.

ALB reflects systemic nutritional status and plays a role in various essential bodily defensive mechanisms 36. In the inflammatory state, microvascular permeability increases and plasma ALB leaks into the tissue space, increasing the distribution volume of ALB. Simultaneously, inflammation can shorten the half-life of ALB and reduce its overall quantity 37. HBV infection and HCC maintain the inflammatory state, with the tumour burden leading to liver function impairment and malnutrition. These factors negatively affect ALB levels. ALB plays an important role in HCC development, serving as a biomarker to monitor systemic inflammatory status and liver function. For example, low ALB levels are strongly associated with more aggressive tumour parameters. Adding ALB to the culture medium of HCC cells slows HCC cell growth, whereas a marked decrease in ALB level promotes HCC invasion and migration 22, 38, 39. Notably, albumin plays a pivotal role in maintaining the normal biconcave shape of red blood cells. Previous studies have demonstrated that albumin significantly reduces the mechanical and osmotic fragility of red blood cells, thereby influencing their volume and geometric structure 40. Consequently, in patients with HBV-HCC, a decline in albumin levels may exacerbate red blood cell morphological abnormalities, indirectly contributing to an increase in RDW levels.

Overall, RAR, as a combined indicator of RDW and albumin, may more sensitively capture the inflammatory status, nutritional condition, and liver function changes in patients with HBV-HCC, thus providing a more accurate prediction of their prognosis.

This study has some limitations. First, although PSM was used to reduce the potential bias of retrospective studies, further prospective studies are warranted to verify this. Second, we only used a single pre-treatment RAR value as a predictor of survival prognosis and did not dynamically observe its changes. Finally, progression-free survival (PFS) time was not included in this study, and the prognostic value of RAR for long-term PFS in patients with HBV-HCC must be investigated.

## Conclusion

RAR, a novel, convenient, and low-cost biomarker, is a potential and valuable prognostic indicator for patients with HBV-HCC that can provide clinicians with valuable insights into tumour burden, inflammation, and nutritional status, making it a promising tool for personalised clinical interventions and prognostic assessments.

## Supplementary Material

Supplementary figures and tables.

## Figures and Tables

**Table 1:** Characteristics of groups before and after propensity-score matching.

Characteristics	Before matching				After matching		
	L-RAR (<0.3217) n = 306	H-RAR (≥0.3217) n = 600	*p* value		L-RAR (<0.3217) n = 209	H-RAR (≥0.3217) n = 209	*p* value
Age (years)	52 [44, 61]	55 [47, 64]	0.002		56 [45, 64]	55 [48, 63]	0.720
Gender			0.804				
Female	41 (13.4%)	84 (14.0%)					
Male	265 (86.6%)	516 (86.0%)					
WBC (×10^9^/L)	5.81 [4.83, 7.32]	5.86 [4.83, 7.32]	0.573				
NEU (×10^9^/L)	3.38 [2.67, 4.59]	3.73 [2.63, 5.21]	0.042		3.51 [2.65, 4.75]	3.37 [2.52, 4.60]	0.392
RBC (×10^12^/L)	4.72 [4.44, 5.03]	4.20 [3.73, 4.74]	<0.001		4.64 [4.33, 4.99]	4.52 [4.01, 5.04]	0.132
HCT (%)	43.40 [40.88, 46.00]	38.60 [34.80, 42.28]	<0.001		42.20 [39.90, 44.85]	42.00 [38.30, 45.20]	0.297
MCV (fL)	92.05 [88.50, 95.20]	93.00 [87.60, 96.80]	0.295				
HBV DNA (IU/mL)			<0.001				0.623
Negative	160 (52.3%)	220 (36.7%)			96 (45.9%)	91 (43.5%)	
Positive	146 (47.7%)	380 (63.3%)			113 (54.1%)	118 (56.5%)	
Cirrhosis			<0.001				0.338
No	110 (35.9%)	117 (19.5%)			59 (28.2%)	68 (32.5%)	
Yes	196 (64.1%)	483 (80.5%)			150 (71.8%)	141 (67.5%)	
HBeAg			0.443				
Negative	241 (78.8%)	459 (76.5%)					
Positive	65 (21.2%)	141 (23.5%)					
Maximum tumor diameter (cm)	4.85 [2.50, 8.50]	8.60 [4.60, 12.48]	<0.001		6.00 [3.30, 10.00]	6.40 [3.35, 10.35]	0.562
Tumor number			<0.001				0.282
Single	184 (60.1%)	225 (37.5%)			109 (52.2%)	98 (46.9%)	
Multiple	122 (39.9%)	375 (62.5%)			100 (47.8%)	111 (53.1%)	
TNM stage			<0.001				0.773
Ⅰ	136 (44.4%)	113 (18.8%)			71 (34.0%)	63 (30.1%)	
Ⅱ	58 (19.0%)	83 (13.8%)			36 (17.2%)	40 (19.1%)	
Ⅲ	85 (27.8%)	261 (43.5%)			75 (35.9%)	74 (35.4%)	
Ⅳ	27 (8.8%)	143 (23.8%)			27 (12.9%)	32 (15.3%)	
Treatment			<0.001				0.519
Operation	272 (88.9%)	368 (61.3%)			175 (83.7%)	170 (81.3%)	
Non-Operation	34 (11.1%)	232 (38.7%)			34 (16.3%)	39 (18.7%)	
Diabetes Mellitus			0.086				
No	282 (92.2%)	531 (88.5%)					
Yes	24 (7.8%)	69 (11.5%)					
Hypertension			0.270				
No	266 (86.9%)	505 (84.2%)					
Yes	40 (13.1%)	95 (15.8%)					

WBC, white blood cell; NEU, neutrophil; RBC, red blood cell; HCT, hematocrit; MCV, mean corpuscular volume; RAR, red blood cell distribution width to albumin ratio

**Table 2:** Univariable and multivariable Cox regression analyses to identify predictors of overall survival after propensity-score matching

	Univariate analysis				Multivariate analysis		
	HR	95% CI	*p* value		HR	95% CI	*p* value
Age	0.982	0.973-0.992	0.001		1.001	0.988-1.013	0.915
Gender							
Male	1.625	1.111-2.375	0.012		1.176	0.771-1.794	0.451
WBC	1.059	1.007-1.114	0.026		0.901	0.414-1.960	0.792
NEU	1.119	1.060-1.182	<0.001		1.143	0.528-2.475	0.734
LYM	0.653	0.536-0.796	<0.001		0.914	0.382-2.188	0.839
Monocyte	2.863	1.628-5.036	<0.001		1.092	0.315-3.787	0.890
RBC	1.066	0.888-1.279	0.494				
HCT	1.020	0.992-1.048	0.159				
MCV	1.008	0.991-1.025	0.341				
PLT	1.002	1.001-1.003	0.007		0.999	0.998-1.001	0.569
RAR							
High	1.707	1.338-2.176	<0.001		1.571	1.202-2.053	0.001
Child-Pugh grade			<0.001				<0.001
B	2.354	1.644-3.370	<0.001		1.298	0.881-1.913	0.187
C	15.493	6.580-36.476	<0.001		8.442	3.353-21.255	<0.001
HBV DNA							
Positive	1.402	1.098-1.790	0.007		1.089	0.836-1.418	0.528
AFP							
≥400	2.137	1.679-2.720	<0.001		1.046	0.781-1.400	0.763
Cirrhosis							
Yes	0.973	0.752-1.259	0.835				
HBeAg							
Positive	1.033	0.772-1.382	0.828				
TNM stage			<0.001				<0.001
Ⅱ	1.870	1.239-2.822	0.003		1.468	0.912-2.362	0.114
Ⅲ	5.077	3.602-7.156	<0.001		2.988	1.797-4.969	<0.001
Ⅳ	10.218	6.835-15.276	<0.001		4.411	2.440-7.974	<0.001
Maximum tumor diameter	1.131	1.105-1.157	<0.001		1.053	1.016-1.092	0.005
Tumor number							
Multiple	2.760	2.149-3.544	<0.001		1.252	0.899-1.743	0.184
Treatment							
Non-operation	3.369	2.536-4.475	<0.001		2.099	1.488-2.960	<0.001
Diabetes Mellitus							
Yes	0.997	0.665-1.494	0.987				
Hypertension							
Yes	0.787	0.549-1.129	0.193				

WBC, white blood cell; NEU, neutrophil; LYM, lymphocyte; RBC, red blood cell; HCT, hematocrit; MCV, mean corpuscular volume; PLT, platelet; RAR, red blood cell distribution width to albumin ratio

**Table 3:** Impact of adding RAR on prognostic model performance

Model	C-index	IDI (%) (95% CI)	*p*-value	NRI (%) (95% CI)	*p*-value	Likelihood ratio chi-square	*p*-value	Time-dependent AUC
BCLC	0.745	Ref	Ref	Ref	Ref	Ref		0.815
BCLC + RAR	0.757	5.0 (2.3-8.6)	<0.001	36.1 (23.4-47.1)	<0.001	21.102	<0.001	0.851
TNM	0.734	Ref	Ref	Ref	Ref	Ref		0.787
TNM + RAR	0.749	6.1 (3.0-10.0)	<0.001	36.1 (25-48.7)	<0.001	28.497	<0.001	0.840
Child-Pugh grade	0.554	Ref	Ref	Ref	Ref	Ref		0.575
Child-Pugh grade + RAR	0.585	4.1 (1.0-7.8)	<0.001	34.1 (21.3-44.0)	<0.001	9.499	0.002	0.698
CLIP	0.533	Ref	Ref	Ref	Ref	Ref		0.572
CLIP + RAR	0.566	5.0 (1.6-9.5)	<0.001	36.1 (22.6-47.9)	<0.001	12.587	<0.001	0.683
ALBI	0.588	Ref	Ref	Ref	Ref	Ref		0.685
ALBI + RAR	0.590	0.8 (-0.4-3.8)	0.259	34.4 (-3.6-45.4)	0.259	0.207	0.649	0.692

IDI, NRI and AUC were calculated at 5 years. BCLC, Barcelona clinic liver cancer; CLIP, cancer of the liver italian program; ALBI, albumin-bilirubin score; RAR, red blood cell distribution width to albumin ratio; C-index, Harrell's concordance index; IDI, integrated discrimination improvement; NRI, net reclassification improvement index; Ref, reference; CI, confidence interval
