# Supplementary material for: The Red Cell Distribution Width to Albumin Ratio: A Novel Prognostic Indicator in Hepatitis B Virus-Related Hepatocellular Carcinoma
Source: Int J Med Sci. 2025 Jan 1;22(2):441–50. doi: 10.7150/ijms.103125 (PMC11704691; doi:10.7150/ijms.103125)
Supplement: Supplementary file 1 — Supplementary figures and tables. [file ijmsv22p0441s1.pdf]

Table S1. Baseline characteristics

Characteristics	Total patients = 906	Characteristics	Total patients = 906
Age (years)	54 [46-63]	Cirrhosis	
Gender		No	227 (25.1%)
Male	781 (86.2%)	Yes	679 (74.9%)
Female	125 (13.8%)	HBeAg	
WBC (×10^{9}/L)	5.82 [4.57-7.42]	Negative	700 (77.3%)
NEU (×10^{9}/L)	3.60 [2.66-4.99]	Positive	206 (22.7%)
LYM (×10^{9}/L)	1.41 [1.02-1.86]	Maximum tumor diameter (cm)	7.30 [3.50-11.35]
Monocytes (×10^{9}/L)	0.40 [0.30-0.53]	Tumor number	
RBC (×10^{12}/L)	4.44 [3.92-4.86]	single	409 (45.1%)
RDW (%)	13.90 [13.10-15.10]	multiple	497 (54.9%)
HCT (%)	40.50 [36.70-44.10]	PVTT	
MCV (fL)	92.70 [88.00-96.20]	No	547 (60.4%)
PLT (×10^{9}/L)	166.00 [114.00-224.00]	Yes	359 (39.6%)
ALT (U/L)	39.30 [26.40-70.70]	TNM stage	
AST (U/L)	52.00 [32.40-102.00]	I	249 (27.5%)
ALP (U/L)	115.20 [83.00-169.00]	II	141 (15.6%)
TBIL (μmol/L)	16.60 [11.50-26.50]	III	346 (38.2%)
ALB (g/L)	39.30 [35.10-43.60]	IV	170 (18.7%)
RAR (%/(g/L))	0.3512 [0.3073-0.4233]	Treatment	
PT (sec)	12.60 [11.80-13.70]	Operation	640 (70.6%)
HBV DNA (IU/mL)		Non-Operation	266 (29.4%)
Negative	380 (41.9%)	Diabetes Mellitus	
Positive	526 (58.1%)	No	813 (89.7%)
Child-Pugh grade		Yes	93 (10.3%)
A	662 (73.1%)	Hypertension	
B	198 (21.9%)	No	771 (85.1%)
C	46 (5.1%)	Yes	135 (14.9%)
AFP (ng/mL)			
<400	514 (56.7%)		
≥400	392 (43.3%)		

WBC, white blood cell; NEU, neutrophil; LYM, lymphocyte; RBC, red blood cell; RDW, red blood cell distribution width; HCT, hematocrit; MCV, mean corpuscular volume; PLT, platelet; ALT, alanine aminotransferase; AST, aspartate aminotransferase; ALP, alkaline phosphatase; TBIL, total bilirubin; ALB, albumin; RAR, red blood cell distribution width to albumin ratio; PT, prothrombin time; AFP, alpha fetoprotein; PVTT, portal vein tumor thrombus

Table S2. Univariable and multivariable Cox regression analyses to identify predictors of overall survival before propensity-score matching

	**Univariate analysis**			**Multivariate analysis**		
	HR	95% CI	*p* value	HR	95% CI	*p* value
Age (years)	0.994	0.988-1.001	0.090	1.005	0.998-1.013	0.173
Gender						
Male	1.146	0.908-1.445	0.250	1.125	0.875-1.446	0.359
WBC ($\times10^9$/L)	1.060	1.026-1.095	0.001	0.711	0.491-1.029	0.071
NEU ($\times10^9$/L)	1.141	1.102-1.180	<0.001	1.484	1.007-2.189	0.046
LYM ($\times10^9$/L)	0.493	0.429-0.567	<0.001	1.027	0.636-1.658	0.912
Monocyte ($\times10^9$/L)	1.140	0.986-1.319	0.078			
RBC ($\times10^{12}$/L)	0.733	0.657-0.819	<0.001	0.986	0.820-1.186	0.882
HCT (%)	0.956	0.944-0.969	<0.001	0.992	0.968-1.017	0.527
MCV (fL)	1.002	0.992-1.012	0.711			
PLT ($\times10^9$/L)	1.001	1.000-1.002	0.190			
RAR (%/g/L)						
High	3.209	2.648-3.888	<0.001	1.567	1.251-1.964	<0.001
Child-Pugh grade			<0.001			<0.001
B	2.714	2.271-3.244	<0.001	1.184	0.959-1.461	0.116
C	8.096	5.878-11.151	<0.001	3.178	2.172-4.650	<0.001
HBV DNA (IU/mL)						
Positive	1.629	1.385-1.916	<0.001	0.998	0.839-1.187	0.980
AFP (ng/mL)						
≥400	2.141	1.829-2.505	<0.001	1.124	0.935-1.352	0.212
Cirrhosis						
Yes	1.582	1.307-1.914	<0.001	1.162	0.943-1.431	0.160
HBeAg						
Positive	1.049	0.873-1.261	0.609			
TNM stage			<0.001			<0.001
II	1.858	1.378-2.506	<0.001	1.519	1.084-2.127	0.015
III	5.755	4.526-7.317	<0.001	2.791	2.011-3.873	<0.001
IV	9.998	7.635-13.094	<0.001	4.096	2.837-5.914	<0.001
Maximum tumor diameter (cm)	1.128	1.113-1.144	<0.001	1.050	1.029-1.072	<0.001
Tumor number						
multiple	2.692	2.280-3.179	<0.001	1.207	0.981-1.484	0.075
Treatment						
Non-operation	4.122	3.488-4.870	<0.001	1.874	1.545-2.273	<0.001
Diabetes Mellitus						
Yes	0.979	0.761-1.260	0.870			
Hypertension						
Yes	1.002	0.806-1.245	0.989			

WBC, white blood cell; NEU, neutrophil; LYM, lymphocyte; RBC, red blood cell; HCT, hematocrit; MCV, mean corpuscular volume; PLT, platelet; RAR, red blood cell distribution width to albumin ratio

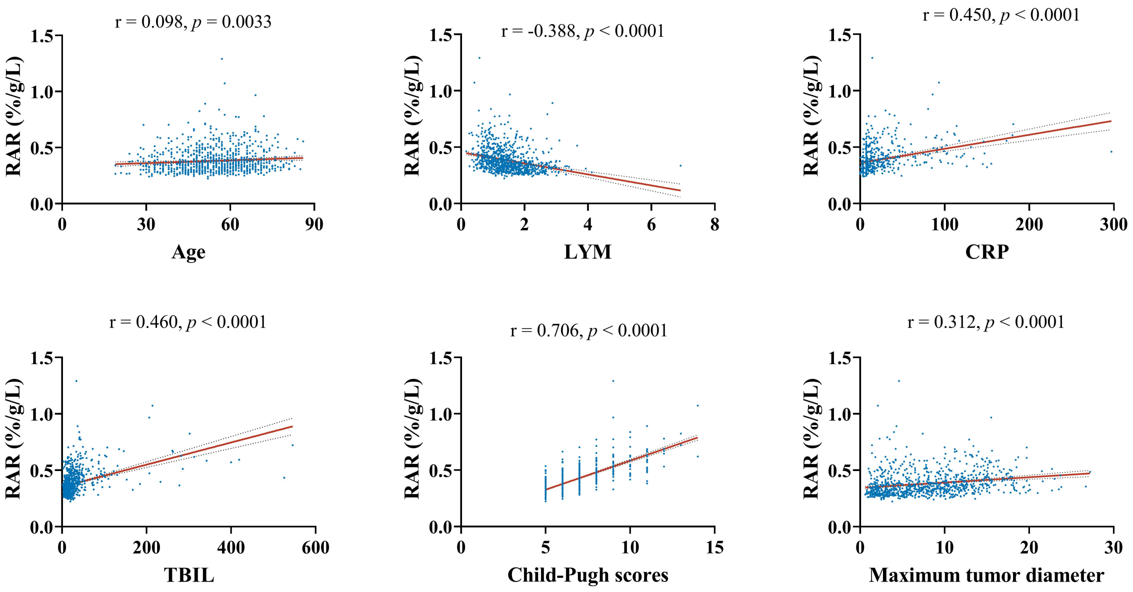

Figure S1. Correlations between RAR with age, LYM, CRP, TBIL, Child-Pugh scores, and maximum tumour diameter in patients with HBV-HCC

RAR, red blood cell distribution width to albumin ratio; LYM, lymphocyte; CRP, C-reactive protein; TBIL, total bilirubin; HBV-HCC, HBV-related hepatocellular carcinoma

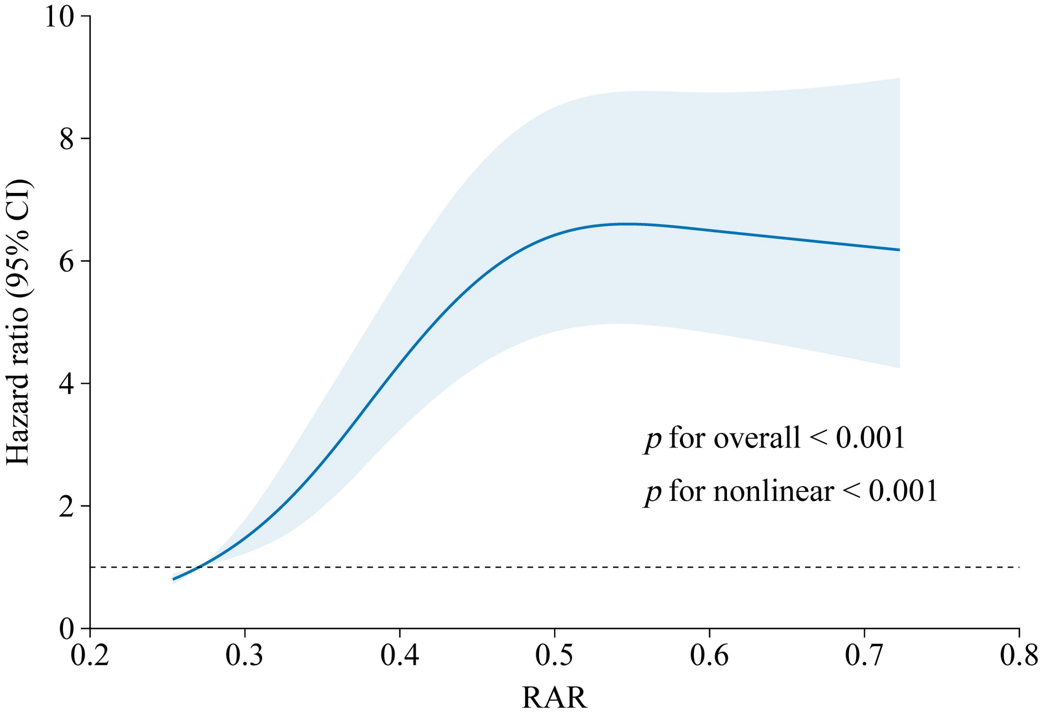

Figure S2. Non-linear relationship between RAR and all-cause mortality risk using restricted cubic spline; y-axis refers to hazard ratio, x-axis refers to RAR value

RAR, red blood cell distribution width to albumin ratio

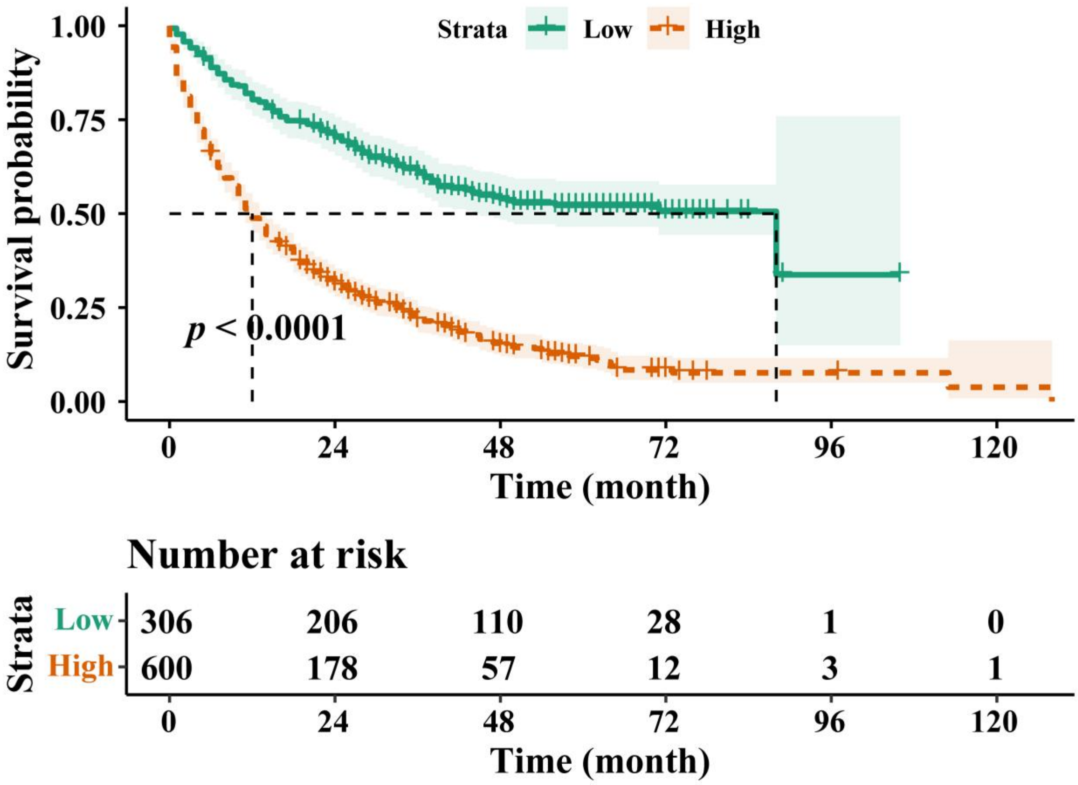

Figure S3. Overall survival stratified by RAR in the entire cohort before propensity-score matching

RAR, red blood cell distribution width to albumin ratio

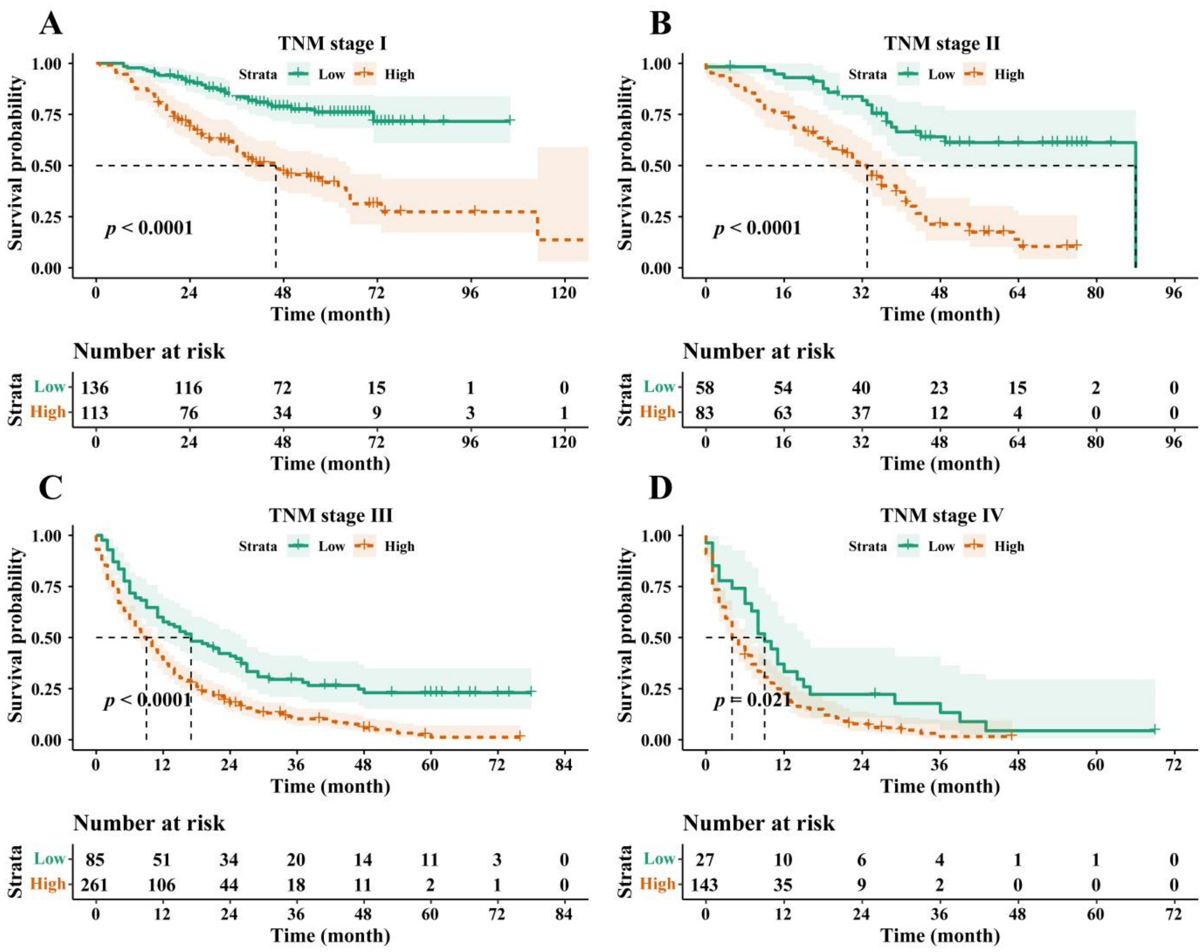

Figure S4. Overall survival stratified by RAR in TNM stage I (A), stage II (B), stage III (C), or stage IV (D) cohorts before propensity-score matching

TNM, Tumour, Node, and Metastasis; RAR, red blood cell distribution width to albumin ratio

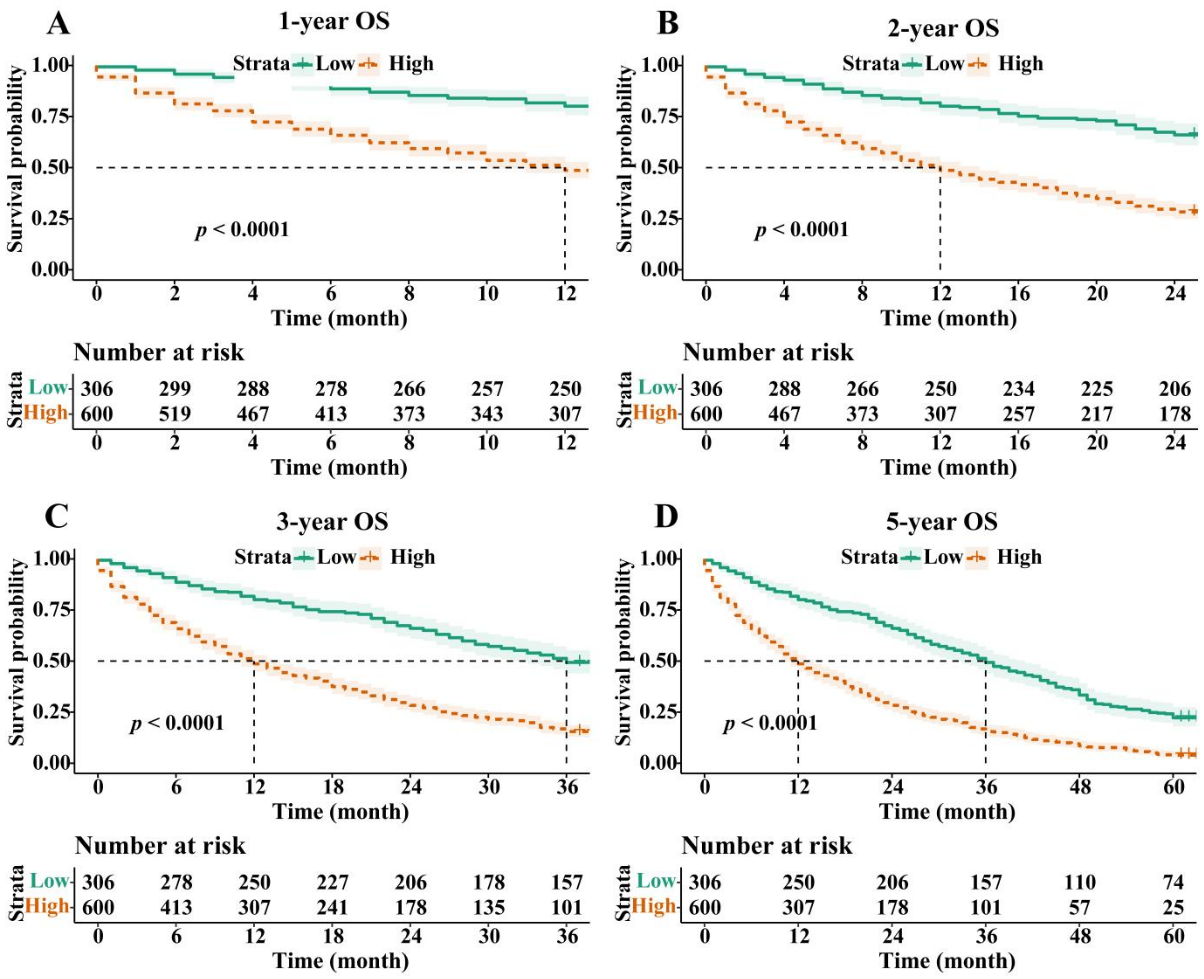

Figure S5. Overall survival stratified by RAR in different periods before propensity-score matching: 1 year (A), 2 years (B), 3 years (C), and 5 years (D)

RAR, red blood cell distribution width to albumin ratio

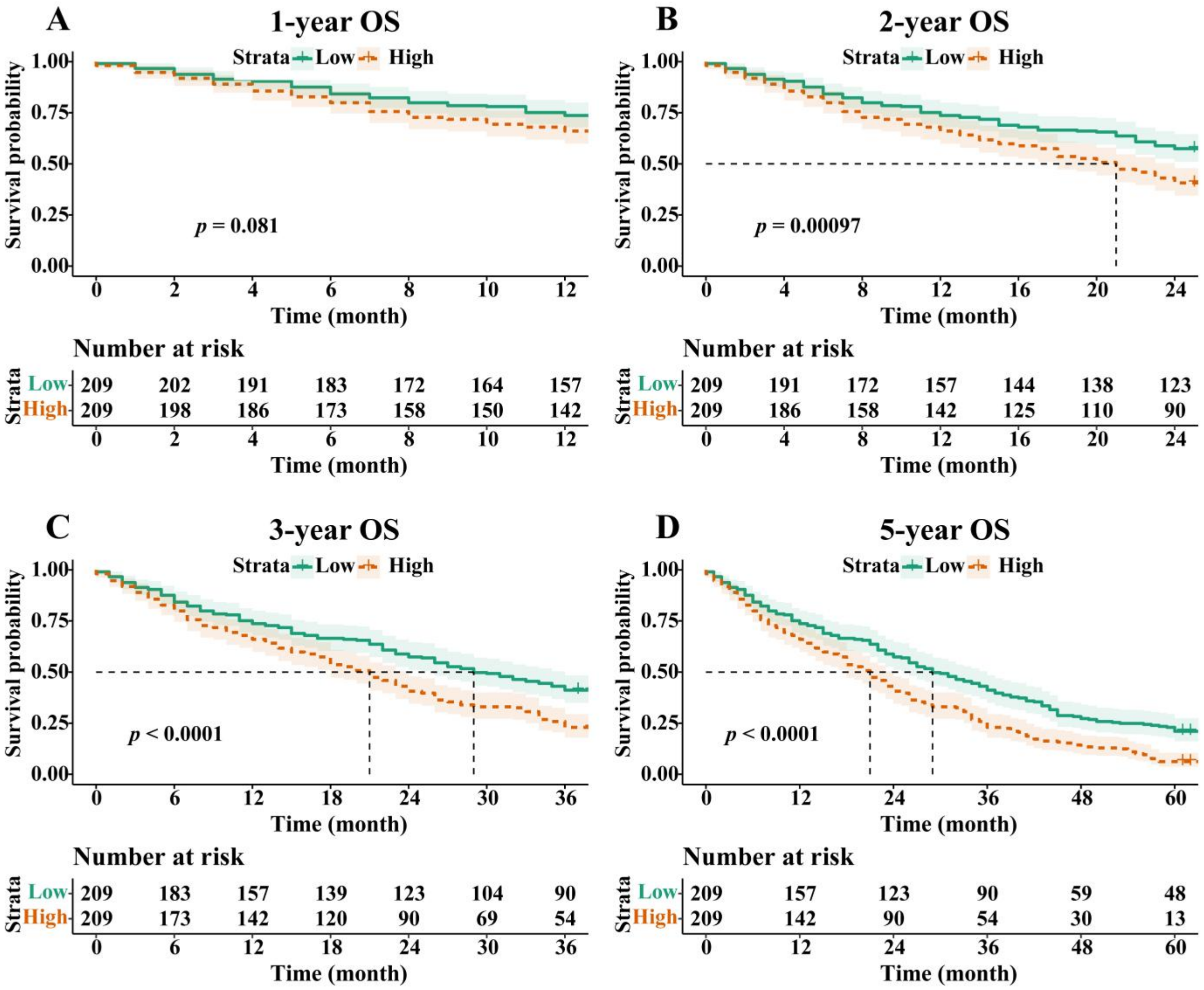

Figure S6. Overall survival stratified by RAR in different periods after propensity-score matching: 1 year (A), 2 years (B), 3 years (C), and 5 years (D)

RAR, red blood cell distribution width to albumin ratio

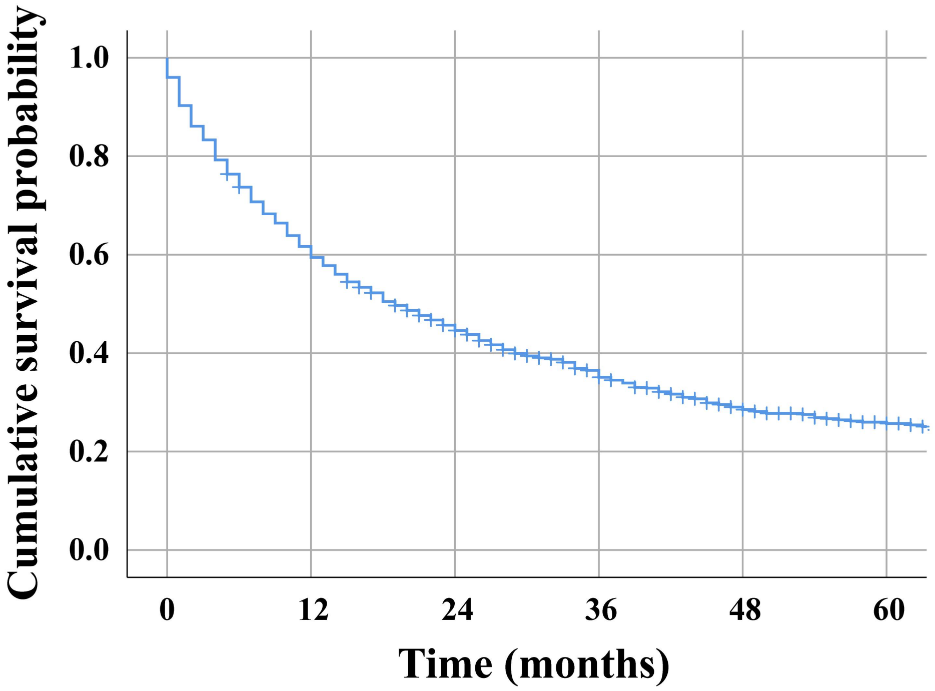

Figure S7. Kaplan-Meier overall survival curve for the entire cohort

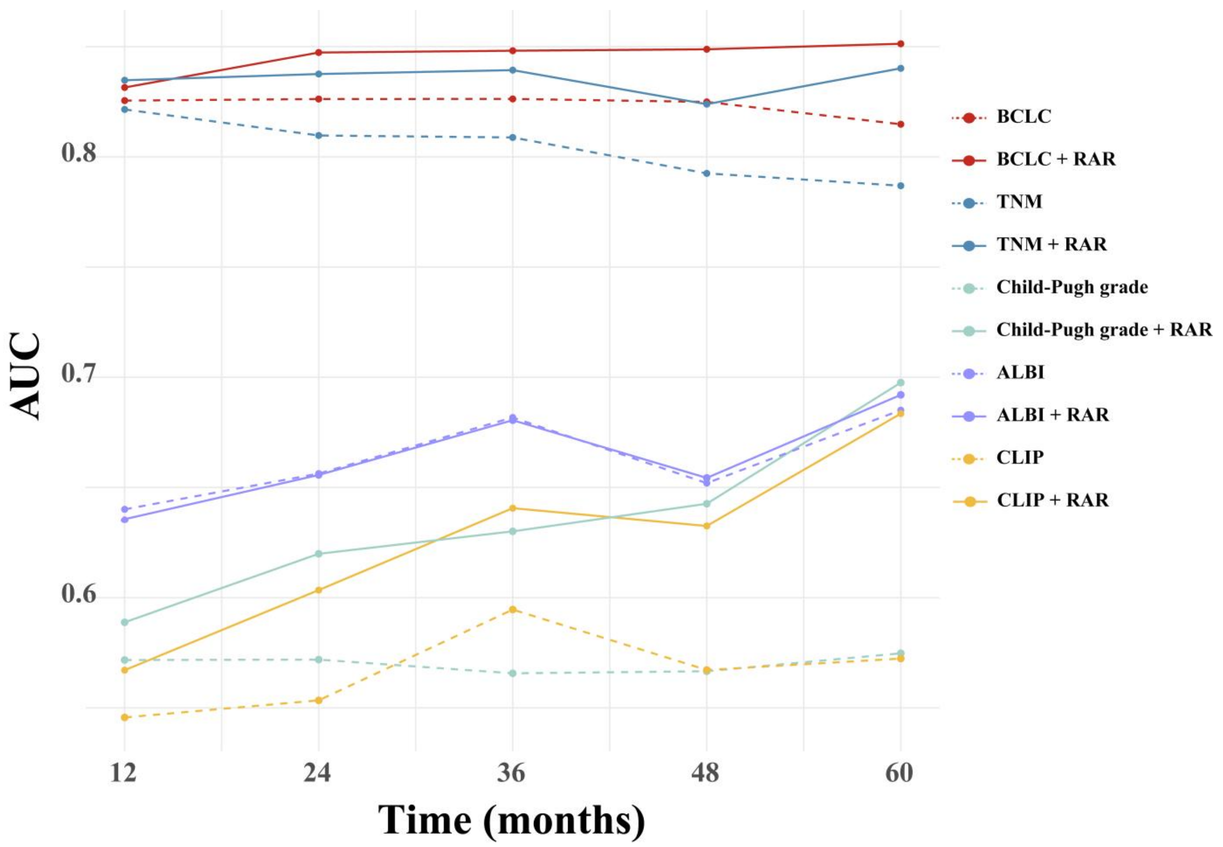

Figure S8. Time-dependent receiver operating characteristics (ROC) curves for the models

BCLC, barcelona clinic liver cancer; RAR, red blood cell distribution width to albumin ratio; TNM, Tumour, Node, and Metastasis staging system; ALBI, albumin-bilirubin score; CLIP, cancer of the liver italian program
